# Editorial: Recent advances in hypospadiology

**DOI:** 10.3389/fped.2023.1226156

**Published:** 2023-11-21

**Authors:** Tariq Abbas, Nicolas Fernandez, Luke Harper

**Affiliations:** ^1^ Department of Urology, Sidra Medicine, Doha, Qatar; ^2^ Department of Pediatric Urology, Seattle Children’s Hospital, Seattle, WA, United States; ^3^ Department of Pediatric Surgery, CHU Pellegrin-Enfants, Bordeaux, France

**Keywords:** hypospadias, genotyping, phenotyping, surgical outcomes, patient reported outcomes

**Editorial on the Research Topic** Recent advances in hypospadiology

Hypospadias is one of the most common genital anomalies in men and displays a wide range of complex phenotypes. Multiple studies have reported a marked global increase in hypospadias cases over the past three decades, although the underlying basis for this remains unclear (1, 2). While hundreds of surgical procedures have been devised to correct hypospadias, only a handful have achieved widespread adoption and acceptable success rates. Unfortunately, high incidence of serious complications persists, partly due to current subjectivity and irreproducibility in case selection and difficulty evaluating different surgical procedures. Indeed, given the diversity of hypospadias phenotypes that can present, it is possible that no single urethroplasty technique will be universally effective. Nonetheless, surgical problems are clearly more common as hypospadias severity increases. Repair dehiscence or persistence of penile curvature may occur, but fistulas and urethral strictures are the most frequent complications that may require further surgery. The scope of this edition is to showcase frontline manuscripts in the field of hypospadiology, addressing major topics including surgical technique and patient-reported outcomes. We thank the authors for their contributions and highlight a number of important results presented in this manuscript collection.

Penile curvature (PC) is a key anatomical component of many hypospadias phenotypes and can have long-lasting effects on patients’ psychosexual health and quality of life. While several prior studies have attempted to establish criteria for evaluating PC, there is still no standardized method for doing so. Despite significant challenges with assessing PC in both clinical practice and research settings, numerous procedures are now being investigated that have potential to resolve longstanding issues. Baray et al., developed an automated deep learning-based method for accurate PC measurement using 2D images, which could significantly improve patient assessment by surgeons and researchers alike. The novel pipeline they propose includes three key consecutive steps: penile localization, shaft segmentation, and angle measurement considering only the proximal and distal parts of the penile shaft. This method may overcome current limitations encountered by conventional approaches to measuring arc-type PC.

Among the wide spectrum of hypospadias anomalies, proximal cases include a subset of severe phenotypes that can significantly impact treatment protocols and post-operative results. In these instances, choice of surgical approach is heavily influenced by surgeon experience and personal preference in addition to patient anatomy (meatal position, urethral plate quality, extent of PC). There is still considerable disagreement about the optimal method for treating proximal hypospadias, but surgeons are increasingly testing new approaches to achieve better patient outcomes. In a study by Zhou et al., one-stage correction of severe hypospadias was achieved using a free preputial tube graft in conjunction with urethral plate urethroplasty and a Buck's fascia integral covering (BFIC) to protect the neourethra and minimize fistula risk. Using an alternative approach, Li et al., report their experience with transverse preputial island flap urethroplasty (TPIFU) in a retrospective cohort study. In 136 patient who underwent single-stage TPIFU, re-operation to address postoperative complications was required in 39% of cases, the majority of which were due to urethrocutaneous fistulas (*n* = 24 patients, 17.6% of the cohort).

Weidler et al., report their results from a multicenter collaboration evaluating the controversy surrounding optimal timing of surgery. In particular, their study focuses on decision making in individuals with differences in sexual development, as well as their caregivers and healthcare providers. Using a semi-structured interview, the authors evaluated potential determinants of successful outcomes in 110 participants. They identify several key themes including; (1) the nature/type of decision being made, (2) person involved in decision making, (3) timing of conversations about surgery, (4) barriers to decision-making surrounding surgery, (5) elements involved in these discussions, and (6) optimal approach to surgical decision-making. Priority was given to children and adolescents with disorders of sexual differentiation to ensure their involvement in all discussions. Tian et al., present the results of a retrospective cohort of 195 patients who underwent hypospadias repair and subsequently developed postoperative fistulas. Patients with recurrent fistulas after initial closure were compared with patients who achieved successful fistula closure, which revealed an association of catheter type used for drainage with local purulent discharge after surgery.

In summary, while many issues remain unresolved in the field of hypospadiology, novel approaches and improved understanding of surgical and patient-reported outcomes will be crucial to the continuing evolution of this challenging field.

## References

[1] Nordenvall AS Frisén L Nordenström A Lichtenstein P Nordenskjöld A. Population based nationwide study of hypospadias in Sweden, 1973 to 2009: incidence and risk factors. J Urol. (2014) 191(3):783–9. 10.1016/j.juro.2013.09.058 24096117

[2] Manson JM Carr MC. Molecular epidemiology of hypospadias: review of genetic and environmental risk factors. Birth Defects Res Part A Clin Mol Teratol. (2003) 67(10):825–36. 10.1002/bdra.10084 14745936

